# Characterization of three glycerol transporters with broad sugar specificities from *Aspergillus niger*

**DOI:** 10.1007/s00253-026-13854-6

**Published:** 2026-05-08

**Authors:** Liinu Nummela, Christina Lyra, Leena Pitkänen, Henry N. Maina, Miia R. Mäkelä

**Affiliations:** 1 Department of Bioproducts and Biosystems, Aalto University, Espoo, Finland; 2 Department of Microbiology, University of Helsinki, Helsinki, Finland; 3 Department of Food and Nutrition, University of Helsinki, Helsinki, Finland

**Keywords:** *Aspergillus niger*, Glycerol, Polyol, Hexose, Transport, Heterologous expression

## Abstract

**Abstract:**

*Aspergillus niger* is a versatile biotechnological workhorse whose generalist lifestyle enables the utilization of a wide range of sugar compounds, including hexoses, pentoses, and polyols. Yet most predicted *A. niger* sugar transporters remain uncharacterized. Here, we characterized three glycerol transporters—GlpA, GlpB, and GlpC—from *A. niger*. We generated single and double *glp* deletion strains for physiological analysis, among which the Δ*glpA* strain showed a clear phenotype with significantly reduced glycerol consumption, indicating that GlpA is essential for glycerol uptake. Furthermore, both growth on glycerol and its uptake were abolished in the Δ*glpA*Δ*glpB* mutant, while the Δ*glpA*Δ*glpC* mutant showed no growth phenotype on solid medium but lacked glycerol consumption in liquid cultures. This suggests that GlpB plays a supportive role in glycerol uptake, whereas GlpC affects it indirectly. Nevertheless, heterologous expression of the *A. niger glp* genes in a sugar uptake deficient *Saccharomyces cerevisiae* strain showed that all three transporters could transport not only glycerol but also D-mannitol, D-sorbitol, and D-xylitol. In addition, GlpC transported the hexose sugars D-fructose, D-glucose, and D-mannose. These results demonstrate the broad specificities of the *A. niger* Glp transporters, highlighting the need for thorough physiological and functional characterization of filamentous fungal sugar transporters. Our findings advance the understanding of polyol and sugar uptake in *A. niger* and provide candidate transporters for strain engineering towards biotechnological upcycling of glycerol.

**Key points:**

• *GlpA is the main glycerol transporter in A. niger under the studied conditions*

• *ΔglpAΔglpB and ΔglpAΔglpC double deletions abolished glycerol uptake in A. niger*

• *GlpA, GlpB, and GlpC have broad polyol specificity, with GlpC also uptaking hexoses*

**Supplementary information:**

The online version contains supplementary material available at 10.1007/s00253-026-13854-6.

## Introduction

The filamentous fungus *Aspergillus niger* has a long history of use as an industrial workhorse for the production of organic acids, especially citric acid, as well as various enzymes, such as amylases and pectinases (Cairns et al. 2018; Meyer et al. 2020). As a species with a generalist lifestyle, *A. niger* can degrade and convert a wide range of sugar compounds, including hexoses, pentoses, and polyols, into energy and biomolecules through diverse sugar metabolic pathways (Li et al. 2022). This metabolic versatility can be exploited to support the transition towards a circular bioeconomy, in which the efficient bioconversion of feedstocks containing different sugars is a crucial factor.

Glycerol is a triol polyol that forms the backbone of phospholipids and triacylglycerols and is therefore a major component of membrane and storage lipids, thus occurring naturally in all fats and oils (Christoph et al. 2006; Klein et al. 2017). In addition, glycerol serves as a carbon source, maintains redox balance, and protects cells from osmotic stress (Klein et al. 2017). Besides its biological significance in living organisms, including *A. niger*, glycerol is widely used in several industries. It is employed, for example, as a low-calorie sweetening agent in food applications, as a moisturizer and softener in cosmetics, and as an additive in pharmaceutical formulations (Chilakamarry et al. 2022). Moreover, crude glycerol is an abundant by product of the bio-diesel industry (Uprety et al. 2017), and thus, an affordable feedstock for microbial cell factories that produce valuable biochemicals and platform compounds, such as hydrogen, ethanol, and citric acid (Chilakamarry et al. 2022; Nicol et al. 2012).

It is essential to understand the import of glycerol into *A. niger* cells to facilitate its efficient use in cell factory applications. The uptake of sugars across the plasma membrane relies on sugar transporters (STs), most of which belong to the Sugar Porter Family of the Major Facilitator Superfamily (MFS) (Saier et al. 2021). Glycerol uptake has been best characterized in the yeast *Saccharomyces cerevisiae*, in which the active transport of glycerol depends on a single protein, the glycerol/H^+^-symporter Stl1p (Ferreira et al. 2005). In addition, glycerol transporters have been experimentally characterized in other yeast species, such as *Candida albicans* (Kayingo et al. 2009) and *Yarrowia lipolytica* (Erian et al. 2022). However, knowledge about glycerol uptake in filamentous fungi is limited, and so far, only one glycerol transporter has been physiologically characterized in the ascomycete fungus *Aspergillus sydowii* (Zeng et al. 2025).

A recent study identified 90 putatively ST-encoding genes from the MFS in *A. niger* genome (Xu et al. 2024), but only 14 of them have been characterized through physiological or functional experiments (Coelho et al. 2013; Jørgensen et al. 2007; Lin et al. 2020; Meng et al. 2023, 2025; Sloothaak et al. 2014, 2015, 2016; Vankuyk et al. 2004). So far, only a single polyol transporter, LatA, which uptakes L-arabitol, has been identified and physiologically characterized in *A. niger* (Meng et al. 2023), whereas the uptake process of other polyols, including glycerol, is still unclear. Another aspect that remains poorly understood is the functional redundancy of polyol transporters. In strain engineering, broad specificity STs that can uptake multiple carbon sources can enable efficient and complete utilization of complex sugar mixtures by increasing nutrient uptake and growth leading to improved performance of fungal cell factories (Jojima et al. 2010). Therefore, detailed knowledge about the proteins involved in the uptake of glycerol in *A. niger* is required to advance the development of industrial strains that efficiently upcycle glycerol to value-added compounds.

In this study, we evaluated the contribution of three putative glycerol transporters from *A. niger*, GlpA, GlpB, and GlpC, on glycerol uptake. These transporters were selected as proteins of interest based on their close phylogenetic relatedness to previously characterized fungal glycerol transporters (Xu et al. 2024). We generated single and double *glp* gene deletion strains of *A. niger* and investigated their impact on growth and uptake capacity using various polyol and sugar compounds as carbon sources both on solid and in liquid media. To assess the functional abilities and substrate range of the Glp proteins, we heterologously expressed them in *S. cerevisiae* IMK1010 strain deficient in sugar uptake (de Valk et al. 2022). We also performed in silico analyses to explore the structural features of the *A. niger* Glp transporters and compared potential substrate-binding sites among fungal glycerol transporters.

## Materials and methods

### Identification and in silico analyses of candidate glycerol transporters

The amino acid sequences of three predicted *A. niger* glycerol transporters (Xu et al. 2024), i.e., NRRL3_00235 (GlpA), NRRL3_00817 (GlpB) and NRRL3_00935 (GlpC), were retrieved from the JGI MycoCosm portal (https://mycocosm.jgi.doe.gov/Aspni_NRRL3_1.home.html). A literature search was conducted to collect experimentally characterized fungal glycerol transporters of which amino acid sequences were obtained from the NCBI or UniProt databases. The protein structures of *A. niger* Glp and *S. cerevisiae* Stl1 transporters were predicted using AlphaFold3 (Abramson et al. 2024) and visualized in PyMOL (The PyMOL Molecular Graphics System, Version 3.0 Schrödinger, LLC, New York City, New York, USA). Transmembrane domains of the *A. niger* Glp transporters were identified using DeepTMHMM 1.0 (Hallgren et al. 2022) and visualized using TMRPres2D (Spyropoulos et al. 2004). Multiple sequence alignment was carried out using MAFFT (Katoh et al. 2019; Kuraku et al. 2013), and the results were visualized with Jalview (Waterhouse et al. 2009).

### Strains, media, and growth conditions

*Escherichia coli* DH5α (Invitrogen, Carlsbad, California, USA) used for plasmid propagation was cultivated in Luria–Bertani (LB) medium containing 100 mg L^−1^ ampicillin (Sigma-Aldrich, Darmstadt, Germany) at 37 °C. All *A. niger* and *S. cerevisiae* strains used in this study are shown in Table 1. *A. niger* strains were grown at 30 °C in Minimal Medium (MM) (de Vries et al. 2004) or Complete Medium (CM; MM, 2 g L^−1^ tryptone, 1 g L^−1^ yeast extract (Neogen, Lansing, Michigan, USA), 1 g L^−1^ casamino acids (MP Biomedicals, Santa Ana, California, USA)) supplemented with 1.22 g L^−1^ uridine (Molekula, Newton Aycliffe, UK) and relevant sugar as carbon source. Media were further supplemented with 1.5% (w/v) agar (Sigma-Aldrich, Darmstadt, Germany) for solid cultivations.

**Table 1:** Fungal strains used in this study

Organism	Strain	Relevant genotype	Reference
*Aspergillus niger*	CBS 138852 N593 Δ*ku70*	*cspA1*, *kusA*::*amdS*, Δ*pyrG*	Alazi et al. 2016
*A. niger*	Δ*glpA*	*cspA1*, *kusA*::*amdS*, Δ*pyrG* Δ*glpA*	This study
*A. niger*	Δ*glpB*	*cspA1*, *kusA*::*amdS*, Δ*pyrG* Δ*glpB*	This study
*A. niger*	Δ*glpC*	*cspA1*, *kusA*::*amdS*, Δ*pyrG* Δ*glpC*	This study
*Saccharomyces cerevisiae*	IMK1010	*MATa ura3-52 LEU2 HIS3 MAL2-8C mal11-mal12::loxP mal21-mal22::loxP mal31-mal32::loxP mph2/3::loxP mph2/3::loxP-hphNT1-loxP suc2::loxP ima1Δ ima2Δ ima3Δ ima4Δ ima5Δ can1::cas9-natNT2 hxt8Δ hxt14Δ gal2Δ hxt4Δ hxt1Δ hxt5Δ hxt3Δ hxt6Δ hxt7Δ hxt13Δ hxt15Δ hxt16Δ hxt2Δ hxt10Δ hxt9Δ hxt11Δ hxt12Δ stl1Δ*	de Valk et al. 2022
*S. cerevisiae*	IMK1010 + GlpA	IMK1010, pUDE453 *glpA*^a^	This study
*S. cerevisiae*	IMK1010 + GlpB	IMK1010, pUDE453 *glpB*^a^	This study
*S. cerevisiae*	IMK1010 + GlpC	IMK1010, pUDE453 *glpC*^a^	This study
*S. cerevisiae*	IMK1010 + Hxt5^b^	IMK1010, pUDE453 *hxt5*^a, b^	This study
*S. cerevisiae*	IMK1010 + empty	IMK1010, pUDE453 empty^a^	This study

^a^All plasmids are listed in Supplementary Table S1

^b^*S. cerevisiae* Hxt5

*S. cerevisiae* IMK1010 strain (de Valk et al. 2022) was used for functional characterization of *A. niger* glycerol transporters. *S. cerevisiae* was grown in Synthetic Medium (SM), supplemented with filter-sterilized (0.45 μm, VWR®, Radnor, Pennsylvania, USA) vitamin solution (Verduyn et al. 1992) at 30 °C. Medium contained an appropriate amount of sugar or 2% (v/v) ethanol (VWR Chemicals, Radnor, Pennsylvania, USA) as a carbon source and was supplemented with 150 mg L^−1^ uracil (Formedium Ltd., Swaffham, UK) for auxotrophic requirements. All media were sterilized at 121 °C for 20 min.

### Construction, protoplast-mediated transformation, and purification of *A. niger* mutant strains

*A. niger* deletion mutants were generated by CRISPR/Cas9 genome editing as described previously (Song et al. 2018). The guide RNA (gRNA) sequences were designed using Geneious Prime 2025.1.2 software (https://www.geneious.com) based on experimentally determined model described by Doench et al. (2014). The gRNAs targeting the desired loci were expressed from plasmids (Supplementary Table S1) generated to delete the *glpA*, *glpB* and *glpC* genes from *A. niger*.

To construct rescue templates, we amplified the upstream and downstream flanking regions of the target genes from *A. niger* genomic DNA using nested PCR with gene specific primers (Supplementary Table S2). Phusion™ High-Fidelty DNA Polymerase (Thermo Scientific, Waltham, Massachusetts, USA) was used for PCR amplification according to the manufacturer’s instructions in this and all other cloning assays in this study. The upstream reverse and downstream forward primers were designed to include a barcode sequence [actgctaggattcgctatcg]. This sequence served as a homologous region for the fusion of DNA fragments in a nested PCR reaction, to produce the linear rescue template (Song et al. 2018). The resulting rescue templates were purified using the NucleoSpin® Gel and PCR Clean-up kit (Macherey-Nagel, Düren, Germany). All primer sequences (Eurofins Genomics, Ebersberg, Germany; Integrated DNA Technologies, Coralville, Iowa, USA) and plasmids used in this study are listed in Supplementary Table S1 and S2, respectively.

Protoplasting and transformation followed the protocols described previously (Kowalczyk et al. 2017; Kusters-van Someren et al. 1991; Storms et al. 2005). The uridine auxotrophic and non-homologous end-joining deficient *A. niger* strain N593 Δ*ku70* (Table 1) was used as a parental strain. Transformations were conducted using 1 μg of ANEp8-Cas9-*pyrG-*gRNA plasmid DNA combined with 5 μg of purified rescue template. For purification, 10 colonies per mutant were selected from MM plates supplemented with 2% (w/v) D-fructose (Thermo Scientific, Waltham, Massachusetts, USA) and streak-purified three times. Auxotrophic strains were cultured in medium supplemented with uridine, whereas counterselection was performed using 5-fluoroorotic acid (Formedium, Swaffham, UK) to target strains harboring *pyrG* marker gene on the self-replicating plasmid. The deletion mutants among purified transformants were confirmed by colony PCR using Phire DNA Polymerase (Thermo Scientific, Waltham, Massachusetts, USA) according to the manufacturer’s instructions.

### Growth profiling of *A. niger* mutant strains

*A. niger* spores were harvested from solid CM medium into ACES buffer (10 mM *N*-(2-acetamido)−2-amino-ethanesulfonic acid, 0.02% Tween 80, pH 6.8; Sigma-Aldrich, Darmstadt, Germany) and counted using a hemocytometer (Fuchs-Rosenthal BRAND™, Wertheim, Germany). Two μl of 5 × 10^5^ ml^−1^ spore solution was inoculated in duplicates on MM agar containing 25 mM D-glucose (Thermo Scientific, Waltham, Massachusetts, USA), D-fructose, D-mannose (Thermo Scientific, Waltham, Massachusetts, USA), sucrose (Sigma-Aldrich, Darmstadt, Germany), maltose (Riedel de Haen, Seelze, Germany), D-xylose (SAFC, Darmstadt, Germany), L-arabinose (VWR Chemicals, Radnor, Pennsylvania, USA), glycerol (VWR Chemicals, Radnor, Pennsylvania, USA), D-mannitol (Sigma-Aldrich, Darmstadt, Germany), D-sorbitol (Sigma-Aldrich, Darmstadt, Germany), L-arabitol (Thermo Scientific, Waltham, Massachusetts, USA), D-xylitol (Sigma-Aldrich, Darmstadt, Germany), ribitol (Thermo Scientific, Waltham, Massachusetts, USA), D-galactitol (Thermo Scientific, Waltham, Massachusetts, USA), or myo-inositol (Tokyo Chemical Industry Co., Tokyo, Japan) as a carbon source. MM agar without added carbon source was used as a control. Biological duplicate cultures were incubated at 30 °C for 7 days and growth was evaluated through visual inspection.

### Construction of *S. cerevisiae* strains expressing *A. nigerglpA*, *glpB*, or *glpC* genes

Synthetic *glpA*, *glpB*, and *glpC* with overlaps compatible with *GFP*-containing pUDE453 plasmid (Marques et al. 2018) were purchased in the pTwist plasmid (Twist Bioscience, South San Francisco, California, USA), amplified (Supplementary Table S1) and cloned into pUDE453 using Gibson Assembly® Cloning kit (New England Biolabs, Ipswich, Massachusetts, USA). Before the assembly, the plasmid backbone was PCR-linearized with primers listed in Supplementary Table S2. The amplified genes as well as the plasmid backbone were digested with *Dpn*I (New England Biolabs, Ipswich, Massachusetts, USA) to remove the vector backbone. The resulting plasmids (Supplementary Table S1) were transformed into *E. coli* DH5α for propagation.

Plasmids (Supplementary Table S1) were extracted from *E. coli* cells using NucleoSpin® Plasmid EasyPure kit (Macherey-Nagel, Düren, Germany) according to manufacturer’s instructions and transformed into *S. cerevisiae* IMK1010 by lithium acetate/single-stranded carrier DNA/polyethylene glycol method (Gietz and Woods 2002). The yeast cells were plated on SM medium lacking uracil for the selection. The localization of the Glp-GFP fusions at the plasma membrane of the recombinant yeast strains was confirmed using both UV light and phase contrast microscopy (Microscope Zeiss Axioscope 2, Zeiss, Oberkochen, Germany) with 100× magnification and Zen lite program (Version 2.6, Zeiss, Oberkochen, Germany).

### *S. cerevisiae* growth experiments

For growth experiments, the recombinant *S. cerevisiae* strains (Table 1) were inoculated from glycerol stocks into 50 ml of SM medium supplemented with 2% (v/v) ethanol and incubated at 30 °C with 200 rpm for 4–5 days to reach an optical density of 2–8 at 660 nm (OD_660_). Cells were washed with SM medium without carbon source or vitamins at 3000 × *g* 5 min at 4 °C.

For spotting assay, cells were resuspended to SM to reach 10^7^ cells corresponding to an OD_660_ of 1. Ten μl of serial dilutions containing an estimated number of cells, 10^6^, 10^5^, 10^4^, and 10^3^, were spotted onto SM plates supplemented with 2% agar (w/v) and 1% glycerol, D-mannitol, D-sorbitol, or D-xylitol, or 0.2%, 2%, or 8% D-glucose, D-fructose, D-mannose, or D-galactose (Sigma-Aldrich, Darmstadt, Germany) as a carbon source, and incubated at 30 °C for 8 days. Due to its ability to passively diffuse across the plasma membrane, 2% ethanol was used as a control carbon source. Growth was monitored by photographing the plates on days 3, 6, and 8.

For liquid growth experiment, cells were resuspended to SM to reach 10^8^ cells corresponding to an OD_660_ of 10. Five μl of cell suspension was inoculated into 195 μl of SM with 0.2% or 2% glycerol, 0.5% or 0.05% D-sorbitol, D-mannitol, or D-xylitol, or 0.2%, 2%, or 8% D-glucose, D-fructose, D-mannose, or D-galactose as a carbon source. Cultures were prepared in 96-well microplates (TC Plate 96 Well, Sarstedt AG & Co., Nümbrecht, Germany) in triplicates, to reach an estimated OD_660_ of 0.25. Initial readings were recorded at timepoint 0 after which the plates were incubated at 1050 rpm shaking (Titramax 1000, Heidolph, Schwabach, Germany) at 30 °C for 4 days, and OD_660_ was measured twice a day using Spark microplate reader (Tecan, Männedorf, Switzerland). Before the measurements, the plates were centrifuged at 2000 × *g* for 1 min and shaken at 1050 rpm for 5 min, excluding the initial measurement. Fluorescence measurements were performed using Synergy H1 microplate reader (BioTek, Winooski, Vermont, USA).

### Polyol consumption in *A. niger* mutant strains

Triplicate 50 ml transfer cultivations of the *A. niger* deletion mutant strains and the reference strain were performed as previously described (Meng et al. 2023). The cultures were supplemented with 25 mM glycerol, or 5 mM D-mannitol, D-sorbitol, L-arabitol, or D-xylitol. In glycerol cultures, 25 mM concentration was utilized, since the reference strain grew well and in high concentration the observable effect could be maximized. However, other polyols were applied at 5 mM concentration, because mycelium clumps, an effect that is not related to the deleted ST-encoding genes, were detected in the liquid cultures of the reference at 25 mM concentration. Culture liquid samples were collected at 0, 4, 22, 28, 46 or 48, 50 or 52, and 120 h, centrifuged at 4 °C, 21,300 × *g* for 30 min, and stored at −20 °C. Before the measurement, samples were filtered through 0.22-μm sterile filters (J.T. Baker®, Radnor, Pennsylvania, USA; Clarify™, Torrance, California, USA) and internal standard of 2-deoxy-D-galactose (Sigma-Aldrich, Darmstadt, Germany), glycerol or L-arabinose was added. The extracellular concentration of the samples from the three batches were analyzed with two HPAEC-PAD (high-performance anion-exchange chromatography with pulsed amperometric detection) instruments. Polyols in the Δ*glpA*, Δ*glpB*, and Δ*glpC* cultivations were determined using an Alliance HPLC (high-performance liquid chromatography) system equipped with a Waters e2695 separation module (Milford, Massachusetts, USA), a 3465 EC detector, and a Carbopac MA-1 (4 × 250 mm i.d., Dionex, Waltham, Massachusetts, USA) column. The eluent was 480 mM NaOH at a flow rate of 0.4 ml min^−1^, and the injection volume was 5 µl. Standards from 0.2 to 0.005 mg ml^−1^ were prepared for glycerol, D-mannitol, D-sorbitol, L-arabitol, and D-xylitol. The extracellular concentration of the polyols from the cultivations of double mutants Δ*glpA*Δ*glpB* and Δ*glpA*Δ*glpC* were analyzed using Dionex ICS-5000 HPAEC-PAD with Dionex CarboPac PA20 column, and the flow rate was 0.38 ml min^−1^. Water was used as eluent, and 0.1 M NaOH was added post-column before the pulse-amperometric detector. The calibration curve for each quantified compound was prepared using solutions covering the concentration range from 0.001 to 0.05 mg ml^−1^. Injection volume was 25 µl. Because the experiment was conducted in three batches and samples were analyzed in two different HPAEC-PAD instruments, we normalized the data by aligning time-zero (T0) polyol concentration of each experiment to the starting polyol concentration (C_ref; 25 mM or 5 mM). For each experiment, the mean of the triplicate T0 measurements (C_T0_strain) was used to caculate a correction factor (s_strain = C_ref/C_T0_strain). This factor was then applied to all measurements of each specific strain. An unpaired two-tailed Welch’s *t*-test was performed to compare whether the concentration of the polyols in the culture liquids of *A. niger* Glp transporter mutant strains differed significantly from those of the reference strain over the cultivation period.

## Results

*A. niger* harbors 90 putative STs categorized into ten clades according to their predicted substrate specificities inferred from the experimentally characterized STs present in each clade (Xu et al. 2024). From the clade with glycerol, L-arabitol, and pentose transporters, we chose three candidates, NRRL3_00235, NRRL3_00817, and NRRL3_00935, from herein GlpA, GlpB, and GlpC, respectively, for further investigation. These STs are phylogenetically closely related to each other and to the characterized yeast glycerol transporters, i.e., *S. cerevisiae* Stl1p (Ferreira et al. 2005) and *Debaryomyces hansenii* DhStl1 (González-Hernández 2010), making them promising candidates for studying the glycerol uptake in *A*. *niger*.

### Combined deletion of *glpA* and *glpB* abolishes the growth of *A. niger* on glycerol

**Fig. 1:**
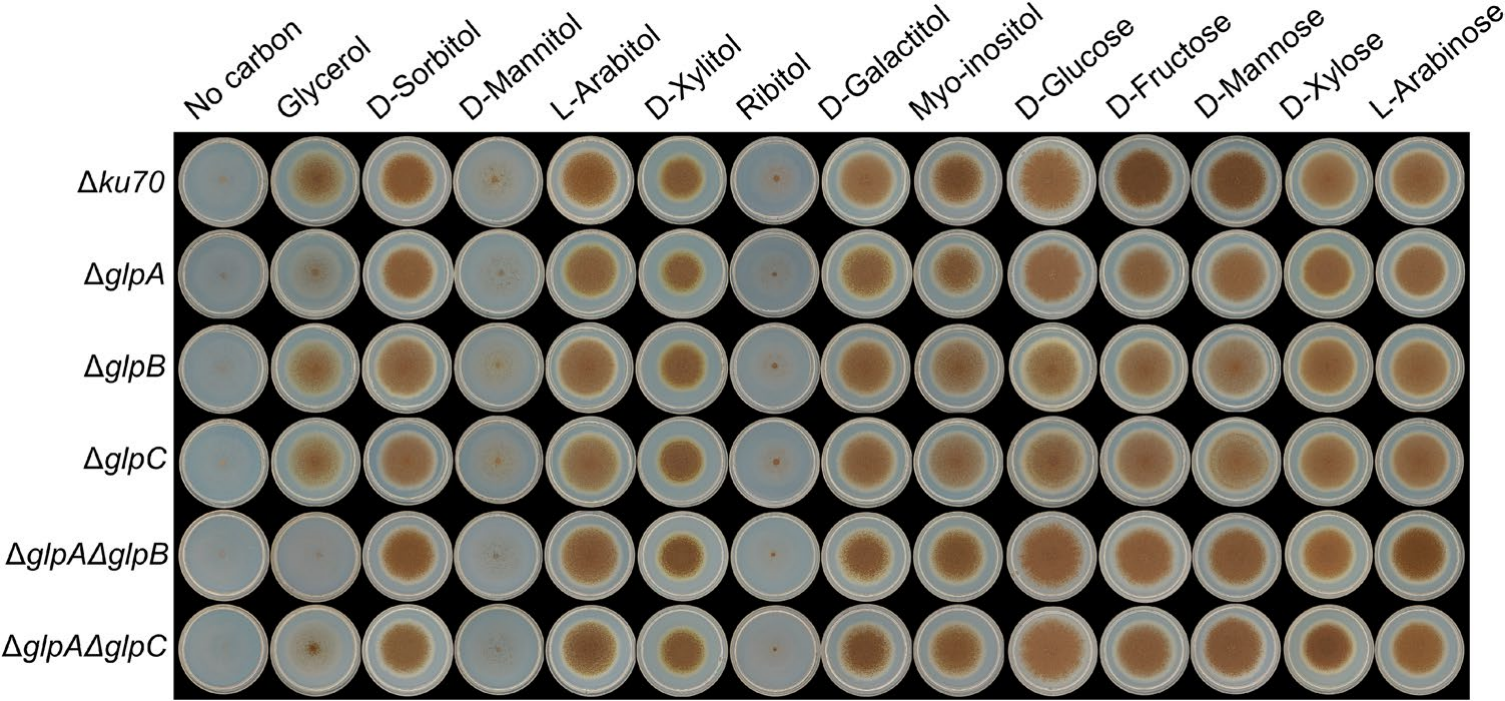
Phenotypic analysis of *Aspergillus niger* Δ*glpA*, Δ*glpB,* Δ*glpC,* Δ*glpA*Δ*glpB*, and Δ*glpA*Δ*glpC* mutants, and the reference strain Δ*ku70* on 25 mM polyols, hexoses, and pentoses. The strains were cultivated at 30 °C for 7 days

To investigate the physiological roles of *glpA*, *glpB*, and *glpC* in *A. niger*, single and double gene deletion mutants were generated of which the Δ*glpA* strain showed clearly reduced growth on glycerol compared to the reference strain (Fig. 1). This demonstrates that GlpA facilitates glycerol uptake under the tested conditions. In contrast, the deletion mutants Δ*glpB* and Δ*glpC* showed no detectable growth defect on glycerol. We generated double deletion mutants of *glpA* together with *glpB* or *glpC* genes to evaluate their effect on *A. niger* phenotype*.* As a result, the growth of the double mutant Δ*glpA*Δ*glpB* was abolished on glycerol (Fig. 1). This indicates that GlpA may have a compensatory role that can partially mask the glycerol uptake ability of GlpB, explaining the absence of a growth phenotype in the Δ*glpB* mutant. However, the phenotype of the double mutant Δ*glpA*Δ*glpC* showed no visible difference from that of Δ*glpA*, suggesting that GlpC may not participate in glycerol uptake under the conditions examined. None of the deletion strains showed phenotypic changes compared to the reference strain when grown on the other tested polyols, hexoses, or pentoses.

### Double deletion mutants Δ*glpA*Δ*glpB* and Δ*glpA*Δ*glpC* show loss of glycerol uptake in *A. niger* liquid cultures

**Fig. 2:**
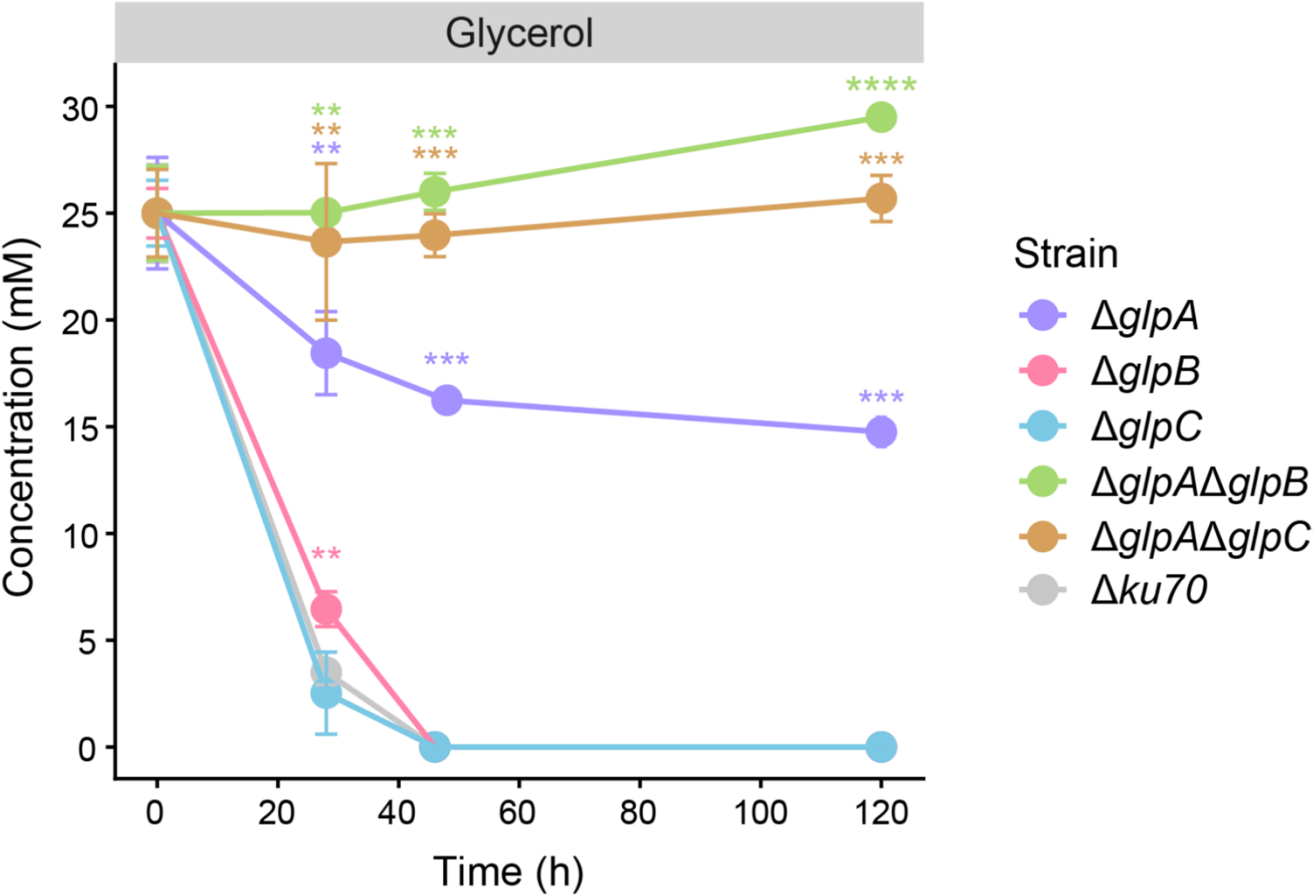
Glycerol consumption of the *Aspergillus niger glp* deletion mutants and the reference strain. Data points represent Δ*glpA* (purple), Δ*glpB* (pink), Δ*glpC* (blue), Δ*glpA*Δ*glpB* (green)*,* Δ*glpA*Δ*glpC* (orange), and Δ*ku70* reference strain (gray). Liquid cultures supplemented with 25 mM glycerol were sampled over time and glycerol concentrations in the supernatants were analyzed using HPAEC-PAD. Due to experiment being conducted in separate runs, all data was normalized using a correction factor determined as the ratio of the starting glycerol concentration and the measured glycerol concentration at 0 h for each experiment. Error bars represent standard deviations from biological triplicates. Statistical significance was determined using an unpaired, two-tailed Welch’s *t*-test with un-equal variances at each timepoint (*P-value ≤ 0.05, **P-value ≤ 0.01, ***P-value ≤ 0.001, ****P-value ≤ 0.0001)

Based on the predicted sugar specificities and the observed phenotypes of the *glp* deletion mutants on solid glycerol medium, we assessed the effects of single and double deletions of the *glp* genes on polyol uptake in *A. niger* liquid cultivations. For this, we compared the consumption of glycerol, D-mannitol, D-sorbitol, L-arabitol, and D-xylitol in the Δ*glpA*, Δ*glpB*, Δ*glpC*, Δ*glpA*Δ*glpB*, and Δ*glpA*Δ*glpC* mutants by determining the polyol concentration in the culture supernatants over time using HPAEC-PAD. As shown in Fig. 2, the Δ*glpA* mutant consumed only 6.6 ± 1.94 mM glycerol within 28 h compared to the reference strain, which consumed most of the glycerol within the same time. After 120 h, 15 ± 0.68 mM glycerol was detected in the culture supernatant of Δ*glpA*. These findings strongly indicate a key role for GlpA in glycerol transport in *A. niger.*

In liquid cultures, the Δ*glpB* strain consumed glycerol slightly slower than the reference strain at 28 h (*P* = 0.009; Fig. 2). This supports the physiological role of GlpB in glycerol uptake in *A. niger*, as indicated by the phenotype of the Δ*glpA*Δ*glpB* double deletion mutant (Fig. 1). No differences in the glycerol uptake were observed between the Δ*glpC* and reference strain under the studied conditions.

As expected, the Δ*glpA*Δ*glpB* mutant did not consume glycerol and approximately 30 ± 0.27 mM glycerol was detected in the liquid medium after 120 h (Fig. 2). Notably, the Δ*glpA*Δ*glpC* double mutant was also unable to consume glycerol in liquid cultures, with 26 ± 1.08 mM glycerol remaining in the medium after 120 h (Fig. 2). These findings further support the glycerol uptake by GlpB and suggest that GlpC may facilitate glycerol uptake indirectly, potentially through regulatory effects that become relevant in the absence of GlpA. The increased glycerol amount in the culture media of the double deletion strains was most probably due to evaporation during incubation which concentrated the growth media. The differences between liquid and solid phenotypes are most likely due to dissimilarities between the growth modes of *A. niger* on solid and in liquid cultivations (Garrigues et al. 2021).

No consistent differences in the uptake of D-mannitol, D-sorbitol, L-arabitol, or D-xylitol were observed between the deletion mutants and the reference strain in liquid cultures. Although Δ*glpA* showed decreased uptake of D-xylitol and Δ*glpB* and Δ*glpC* showed increased uptake of D-mannitol (Supplementary Fig. S1), these observations were not reproducible and may be influenced by differences in mycelial aggregation during the experiments.

### Expression of *glpA*, *glpB*, or *glpC* in *S. cerevisiae* results in growth on polyols and hexoses

**Fig. 3:**
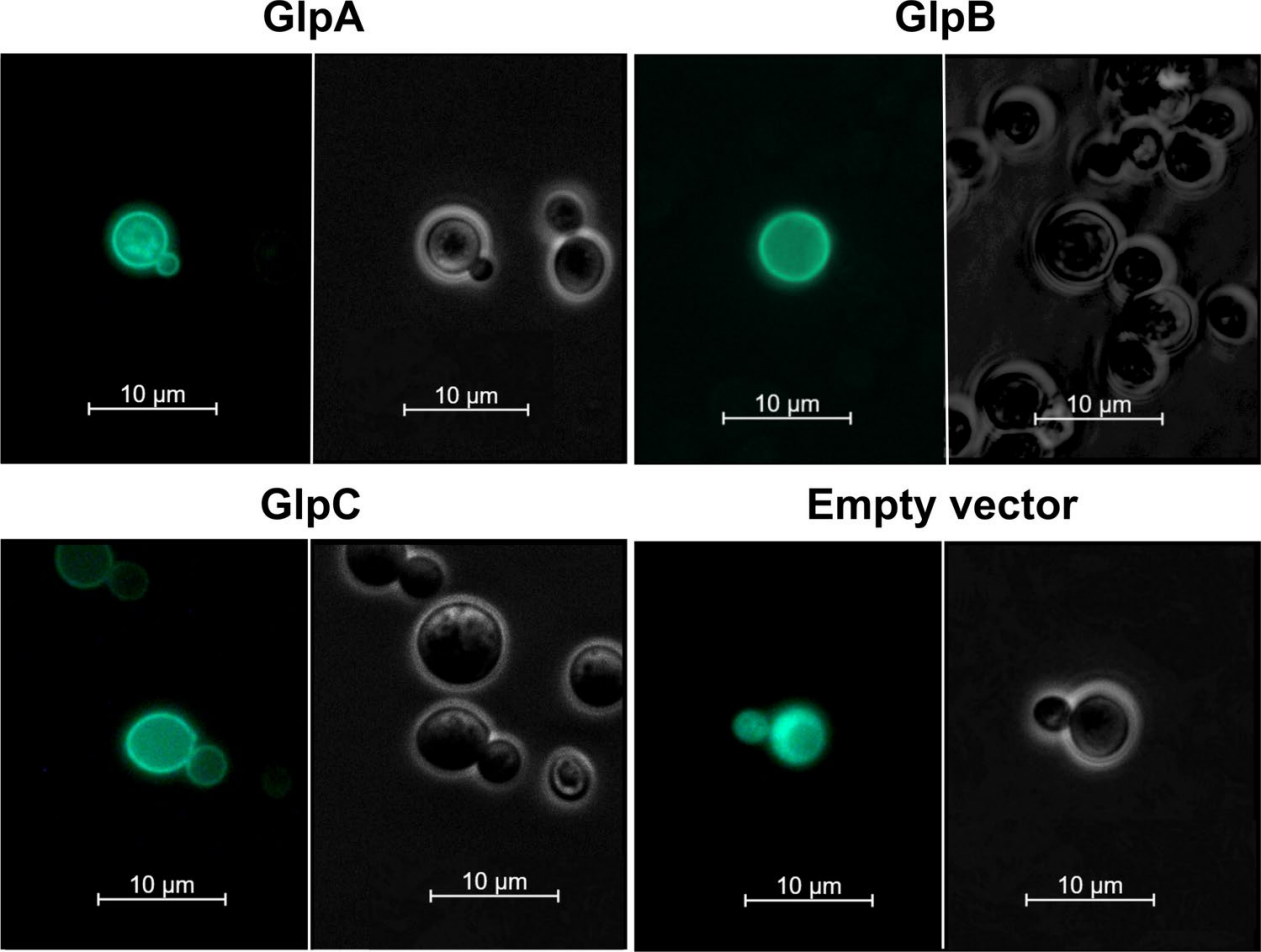
Fluorescent and phase-contrast micrographs showing the localization of *Aspergillus niger* GlpA, GlpB, and GlpC transporters in *Saccharomyces cerevisiae* IMK1010 strain. The correct localization of the transporter at the plasma membrane is indicated by a green halo around the *S. cerevisiae* cell. A strain expressing an empty pUDE453 vector was used as a negative control. Scale bar = 10 μm

To investigate the functional capacity of *A. niger* GlpA, GlpB, and GlpC transporters in vitro, the corresponding genes were heterologously expressed in *S. cerevisiae* IMK1010, a strain lacking all endogenous STs and disaccharide hydrolases (de Valk et al. 2022). Fluorescence microscopy of *S. cerevisiae* strains expressing transporter-GFP fusion proteins demonstrated that all three transporters produced a green halo around the yeast cells (Fig. 3), confirming their correct localization at the plasma membrane*.* The negative control strain expressing empty vector showed no clear fluorescence at the plasma membrane.

**Fig. 4:**
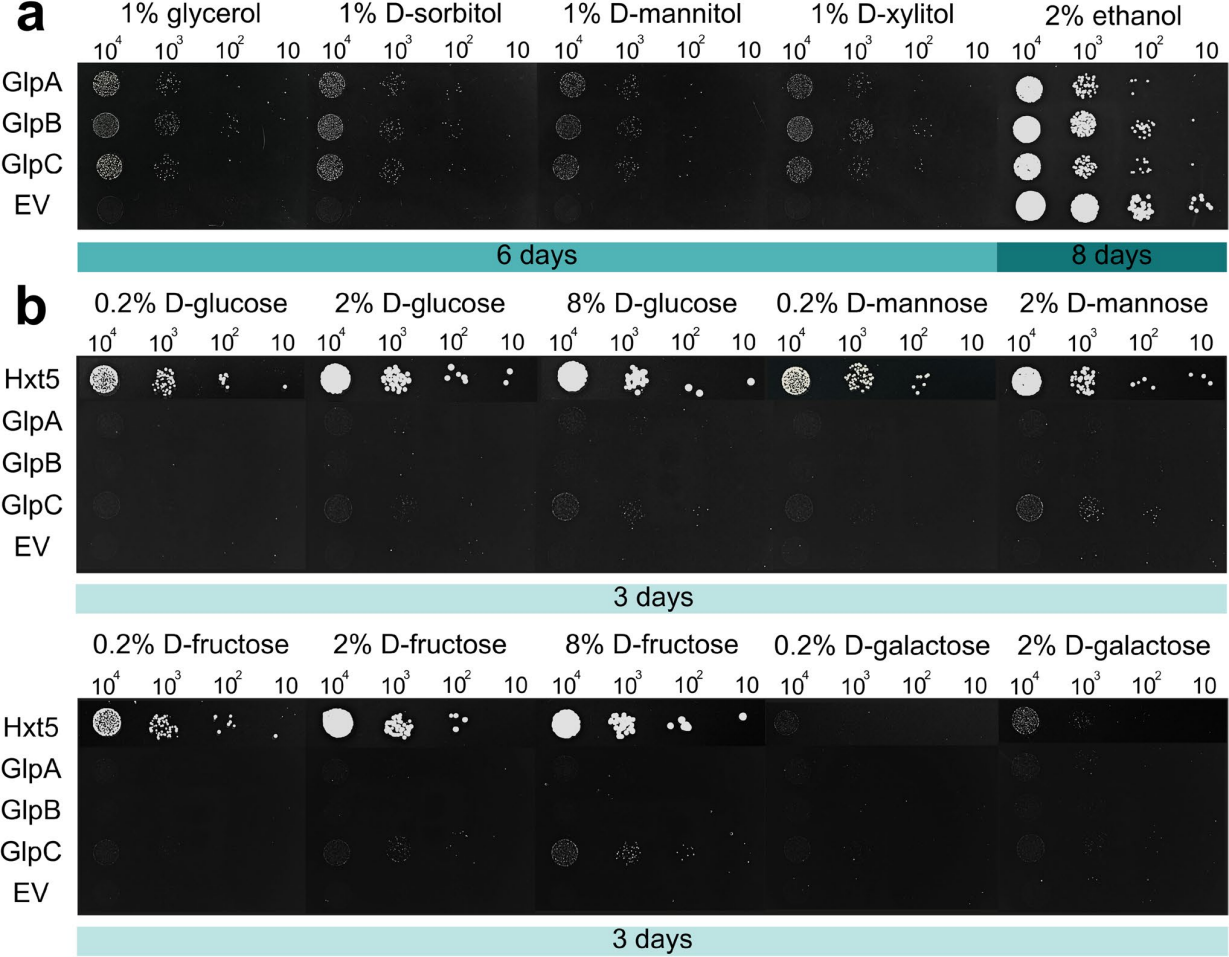
*Saccharomyces cerevisiae* IMK1010 strains producing *Aspergillus niger* GlpA, GlpB, or GlpC transporters. Strains producing the transporters were cultivated as tenfold serial dilutions on synthetic medium supplemented with **a** 1% polyols and **b** 0.2%, 2%, or 8% hexoses. *S. cerevisiae* IMK1010 strain producing endogenous Hxt5 was used as a positive control for growth on hexoses and strain expressing the pUDE453 empty vector (EV) was used as a negative control for sugar uptake. Ethanol was used as a control carbon source for growth

**Fig. 5:**
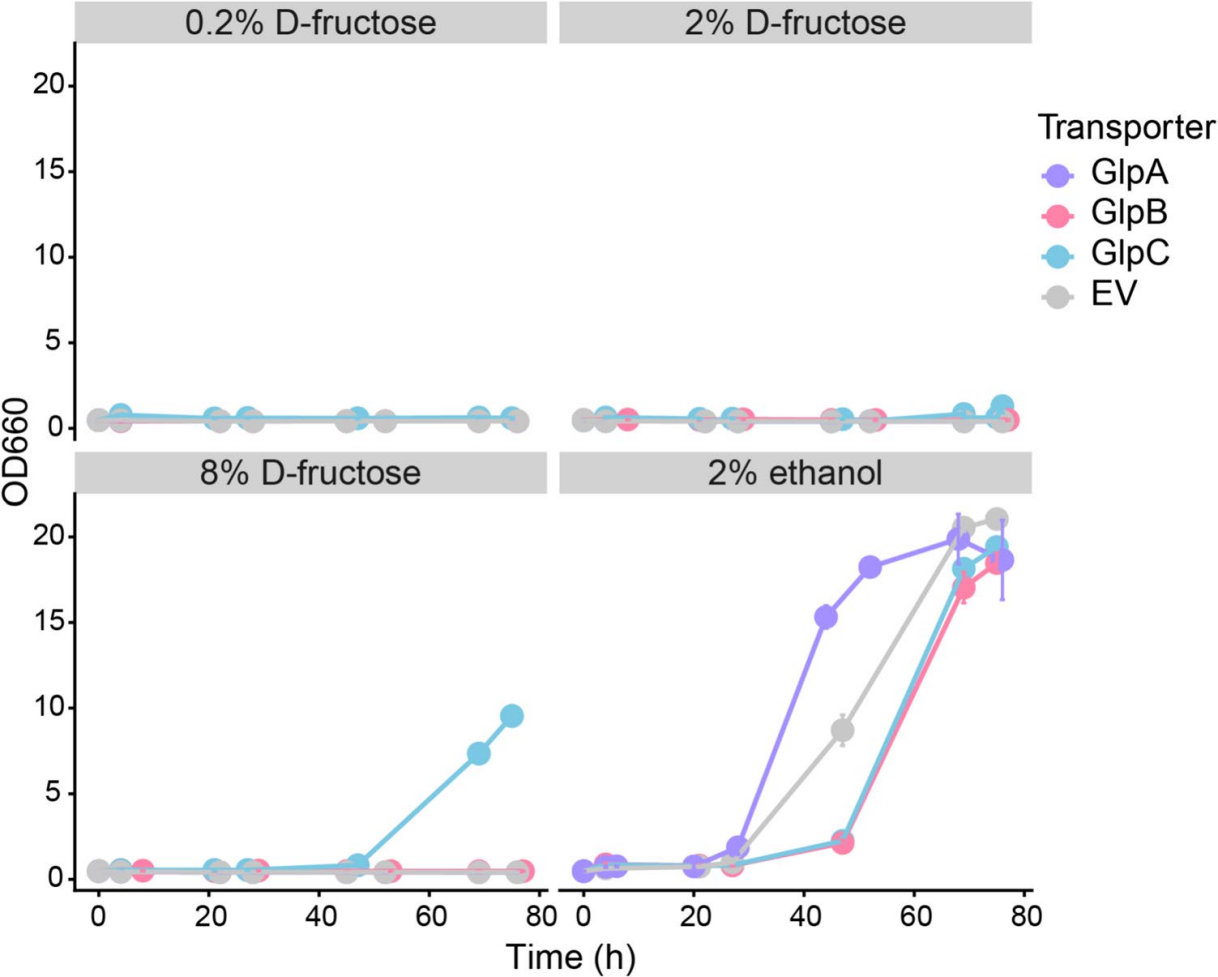
Liquid growth experiment of *Saccharomyces cerevisiae* strains producing *Aspergillus niger* GlpA (purple), GlpB (pink), or GlpC (blue) transporters. D-fructose at different concentrations was used as the carbon source. Strain expressing empty pUDE453 vector (EV; gray) was used as a negative control for sugar uptake. Ethanol was used as a control carbon source for growth. Error bars represent standard deviations from biological triplicates

For functional characterization, we assessed the growth of the *S. cerevisiae* IMK1010 strains producing *A. niger* GlpA, GlpB, or GlpC on solid and in liquid cultures with different polyols or hexoses as carbon sources. On solid media, all *glp*-expressing *S. cerevisiae* IMK1010 strains showed at least slight growth on 1% glycerol, D-mannitol, D-sorbitol, and D-xylitol compared to the negative control after six days of cultivation (Fig. 4a). This indicates the functional ability of all *A. niger* Glp proteins, including GlpC, to transport these polyols into the yeast cells by restoring the growth of the sugar transporter deficient IMK1010 strain. Although the Δ*glpC* strain did not show any phenotype or reduction in uptake with the tested polyols in physiological characterization (Figs. 1 and 2), surprisingly, the yeast strain expressing *glpC* was able to grow on polyols. In addition, the strain grew on 8% D-fructose, 8% D-glucose, and 2% D-mannose (Fig. 4b), which demonstrates a broader sugar specificity of GlpC extending from polyols to hexoses. Only the *S. cerevisiae* strain expressing *glpC* grew in liquid cultures supplemented with 8% D-fructose (Fig. 5, Supplementary Fig. S2), suggesting that GlpC may act as a low-affinity D-fructose transporter. This functional redundancy may indicate wider physiological roles for the Glp transporters in *A. niger* under specific growth conditions. Furthermore, fluorescence microscopy and measurement indicated that the GlpA, GlpB, and GlpC were produced at similar level in liquid cultures supplemented with 2% ethanol (Supplementary Fig. S3).

### Glp transporters share structural and sequence similarities with other characterized fungal glycerol transporters

**Fig. 6:**
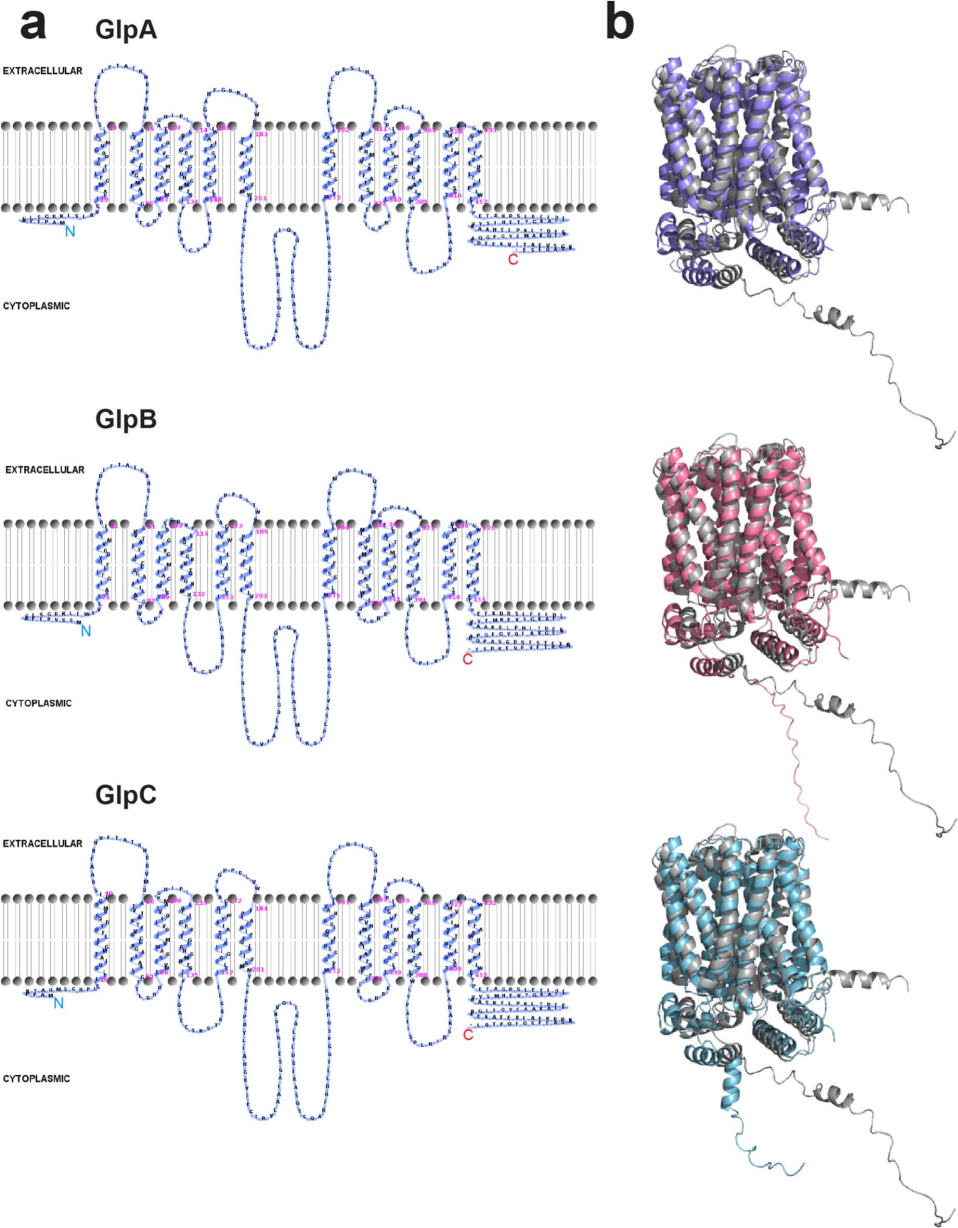
Predicted structures of *Aspergillus niger* GlpA, GlpB, and GlpC transporters. **a** Membrane topology of GlpA, GlpB, and GlpC was predicted using DeepTMHMM (Hallgren et al. 2022) and visualized using TmrPres2D (Spyropoulos et al. 2004). **b** Superimposition of AlphaFold3 predicted structures of *Saccharomyces cerevisiae* Stl1p (gray) with GlpA (purple), GlpB (pink), or GlpC (blue), visualized in PyMOL (The PyMOL Molecular Graphics System, Version 3.0 Schrödinger, LLC)

To gain an insight into the structural features and potential substrate specificity of the *A. niger* Glp transporters, a set of in silico analyses were performed. Topology predictions using DeepTMHMM v1 (Hallgren et al. 2022) indicated that all three transporters have 12 transmembrane domains (Fig. 6a, Supplementary Fig. S4), consistent with typical topology of the MFS transporters (Saier et al. 2021). The α-helical transmembrane domains were connected by hydrophilic loops, and both N- and C-terminal tails were facing the cytoplasm in all three Glp transporters. Each transporter protein primary structure featured a total length ranging from 520 to 540 amino acids. Superimposition of AlphaFold3 structural models of the *A. niger* Glp transporters with that of *S. cerevisiae* Stl1p revealed strong conservation of the transmembrane helices (Fig. 6b). GlpB most closely resembled *S. cerevisiae* Stl1p (root-mean-square deviation, RMSD = 1.636 Å), but GlpC and GlpA also showed high structural similarity (RMSD = 1.826 Å and 2.012 Å, respectively) with Stl1p, which supports their function as glycerol transporters. The greatest differences were observed in the C-terminal tails, which were predicted with the lowest confidence by AlphaFold, likely reflecting their structural flexibility common to cytoplasmic termini of transporters (Mikros and Diallinas 2019). Because both the *A. niger* Glp and *S. cerevisiae* Stl1p models are AlphaFold3 predictions that may be influenced by the existing PDB (Protein Data Bank) templates, the structures and their RMSD comparisons should be interpreted as indicative.

**Fig. 7:**
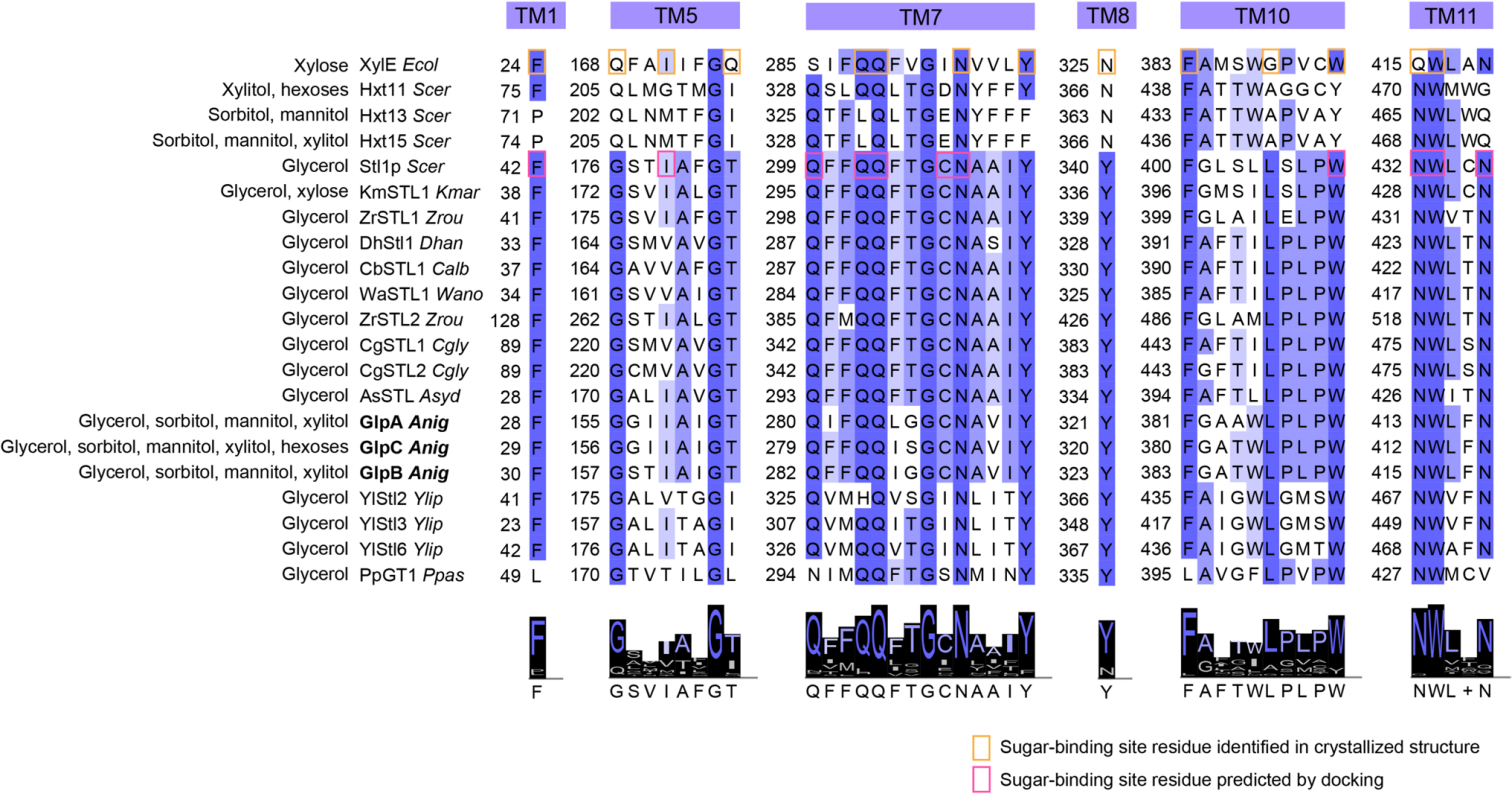
Multiple sequence alignment of sugar transporters and their amino acid residues putatively related to sugar-binding. The alignment included predicted *Aspergillus niger* glycerol transporters GlpA, GlpB, and GlpC, as well as other characterized fungal glycerol transporters (González-Hernández 2010; da Cunha et al. 2019; Dušková et al. 2015; Erian et al. 2022; Ferreira et al. 2005; Ji et al. 2018; Kayingo et al. 2009; Zeng et al. 2025; Zhan et al. 2016; Zhang et al. 2020) and *Saccharomyces cerevisiae* polyol transporters (Jordan et al. 2016). Crystallized *Escherichia coli* XylE xylose transporter (Wisedchaisri et al. 2014) served as a reference to map predicted structures onto the amino acid alignment. The characterized sugar specificities of the transporters are shown on left. *A. niger* GlpA, GlpB, and GlpC are indicated in bold. Alignments were performed using MAFFT v7 (Katoh et al. 2019; Kuraku et al. 2013) and visualized in Jalview v2 (Waterhouse et al. 2009). At the top are indicated the transmembrane helices (TMs) to which the shown amino acids belong. Orange squares indicate sugar-binding residues identified in the crystallized XylE structure. Pink squares indicate putative sugar-binding residues predicted by molecular docking of glycerol to the AlphaFold-predicted model of *S. cerevisiae* Stl1p (Liu et al. 2025). The consensus sequence at the bottom line represents the most frequent amino acid in each alignment position. *Ecol*, *E*. *coli; Scer*, *Saccharomyces cerevisiae*; *Kmar*, *Kluyveromyces marxianus*; *Zrou*, *Zygosaccharomyces rouxii*; *Dhan*, *Debaryomyces hansenii*; *Calb*, *Candida albicans*; *Wano*, *Wickerhamomyces anomalus*; *Cgly*, *Candida glycerinogenes*; *Asyd*, *Aspergillus sydowii*; *Anig*, *Aspergillus niger*; *Ylip*, *Yarrowia lipolytica*; *Ppas*, *Pichia pastoris*

To investigate the putative sugar-binding residues of the *A. niger* GlpA, GlpB, and GlpC, we performed sequence alignment with other characterized fungal glycerol transporters and polyol transporters (Fig. 7). Putative sugar-binding residues, identified through docking analysis of the AlphaFold3-predicted structure of *S. cerevisiae* Stl1p (Liu et al. 2025), were highly conserved across the majority of fungal glycerol transporters, including *A. niger* Glp transporters, suggesting functional conservation of the substrate-binding sites. Although the predictions highlight conserved residues as potential binding sites (Fig. 7), experimental validation, such as site-directed mutagenesis, is required to confirm their functional roles in *A. niger* Glp proteins.

## Discussion

In this study, we physiologically and functionally characterized three putative glycerol transporters—GlpA, GlpB, and GlpC—from the ascomycete fungus *A. niger*, which is commonly used workhorse in biotechnological applications (Meyer et al. 2020). Single deletion of *glpA* led to diminished growth on glycerol and reduced glycerol uptake significantly, whereas the double deletion of *glpA* and *glpB*, abolished both the growth on glycerol and its uptake. In addition, the single deletion of *glpB* led to a statistically significant decrease in glycerol uptake in liquid cultures. Together, these findings suggest that GlpA is primarily responsible for glycerol transport in *A. niger*, whereas GlpB contributes to it to a lesser extent.

The deletion of *glpB* alone did not affect the phenotype of *A. niger* on solid medium, which indicates that GlpA likely compensates the loss of GlpB. This shows that the two STs are functionally redundant under the tested conditions. Comparable findings have been observed in the yeast *Y. lipolytica*, where the presence of several STs with overlapping substrate specificities masks the physiological impact of deleted transporter genes (Erian et al. 2022).

The Δ*glpC* strain grew similarly and consumed glycerol with equal efficiency as the reference strain, indicating that under the tested conditions GlpC is not required for glycerol uptake in *A. niger* when GlpA is present. Moreover, the Δ*glpA*Δ*glpC* double mutant exhibited a phenotype similar to that of Δ*glpA.* In contrast, in liquid culture, the Δ*glpA*Δ*glpC* strain was unable to consume glycerol over the studied time frame, causing more pronounced effect than the single deletion of *glpA*. Our results indicate that the physiological significance of GlpC becomes apparent only in the absence of the major transporter GlpA.

These observations suggest that GlpC or GlpB is required to induce more efficient glycerol metabolism than GlpA alone. Previously, it was shown that deletion of single transporter causes a growth defect in the filamentous ascomycete *Neurospora crassa* (Znameroski et al. 2014). However, only after double transporter deletion, the fungus was unable to induce expression of metabolic genes in response to sugar-based carbon sources. Overall, our *A. niger* double transporter deletion strains show, similarly to those of *S. cerevisiae* (Almeida et al. 2021), that deleting only one transporter may not reveal its full physiological role, but removal of two transporters can unveil additional functions, for example in regulation.

Functional characterization of GlpA, GlpB, and GlpC in *S. cerevisiae* showed that on solid media, all three *A. niger* transporters were not only able to transport glycerol but also D-mannitol, D-sorbitol, and D-xylitol. Furthermore, GlpC transported the hexose sugars D-fructose, D-glucose, and D-mannose. Supporting this substrate range, *glpC* has been shown to be highly expressed (≥ 100 FPKM; fragments per kilo-base of transcript per million mapped reads) in *A. niger* liquid cultivations supplemented with 25 mM D-glucose, D-fructose, D-mannose, and D-xylose after 2 h induction (Xu et al 2024). The stronger growth of the *S. cerevisiae* strain expressing *glpC* with 2% and 8% hexoses indicates that GlpC is likely a low-affinity ST, capable of transporting hexoses more efficiently in higher concentrations. These findings indicate that *A. niger* Glp transporters have broader substrate specificity to polyols and hexoses, similarly to *S. cerevisiae* Hxt hexose and polyol transporters (Jordan et al. 2016).

To the best of our knowledge, none of the previously characterized fungal glycerol transporters have been reported to transport any polyols other than glycerol or monosaccharides. The yeast transporter *Kluyveromyces marxianus* KmSTL1 is the only exception in its ability to transport a pentose sugar D-xylose alongside with glycerol (Donzella et al. 2021). In contrast, the yeast transporters *C. albicans* CbSTL1 and *D. hansenii* DhStl1 have been noted for their specificity to glycerol (Kayingo et al. 2009; Pereira et al. 2014). However, the comparison of amino acid sequences of *A. niger* Glp transporters with previously characterized fungal glycerol transporters revealed that the predicted sugar-binding residues (Liu et al. 2025) were mostly conserved in the selected STs. This suggests that the differences in substrate range of Glp transporters are not reflected at the amino acid sequence level. Sequence similarity in the substrate-binding site region likely explains a common function in glycerol uptake across the so-far characterized fungal glycerol transporters. Plant biomass degrading filamentous fungi, such as *A. niger*, release a mixture of sugars during the degradation process (Mäkelä et al. 2014; Rytioja et al. 2014). Thus, these fungi may benefit from broader sugar specificity of STs, which allows efficient uptake of different sugar compounds and their conversion to energy and biomolecular building blocks through metabolism.

In liquid cultures, *S. cerevisiae* strains expressing Glp transporters did not grow on the tested polyols or hexoses—except fructose—but grew well on ethanol. This suggests that carbon supply via ethanol is not limiting, as it readily diffuses across the plasma membrane, whereas uptake of sugar-based carbon sources may be restricted under the initially low-cell-density conditions in liquid cultures. A plausible explanation can be that the heterologously expressed transporters are not maintained at the plasma membrane (Sen et al. 2016) under the used growth conditions.

Nevertheless, the observation is consistent with the weak growth of the recombinant yeast strains on solid media, which suggests that especially polyols are generally challenging substrates for yeast to metabolize. The *S. cerevisiae* genome encodes several dehydrogenases, including D-mannitol, D-sorbitol, and D-xylitol dehydrogenases (Quain and Boulton 1987; Richard et al. 1999; Sarthy et al. 1994), which oxidize D-mannitol and D-sorbitol to D-fructose and D-xylitol to D-xylulose, respectively. These dehydrogenases enable yeast to grow on the related polyols, although the growth requires good aeration and is typically slow (Quain and Boulton 1987; Richard et al. 1999; Sarthy et al. 1994). The yeast strains deficient in sugar uptake, including *S. cerevisiae* IMK1010 (de Valk et al. 2022), are valuable tools allowing the functional studies of heterologous STs. However, the experimental work could be further facilitated by improving the metabolic abilities of these strains through introduction of additional genes encoding specific metabolic enzymes, such as polyol dehydrogenases.

In summary, this study presents three new glycerol transporters from *A. niger* and demonstrates that GlpA is a main glycerol transporter under the studied conditions. Our findings also highlight the previously unrecognized broader substrate specificity of filamentous fungal glycerol transporters, which extends to other polyols and hexoses. Moreover, Glp transporters could be tested as candidates to optimize the carbon source utilization in *A. niger* cell factories, similarly to *Candida glycerinogenes* CgSTL1 and CgSTL2, and *S. cerevisiae* Stl1p, which have been reported to increase the glycerol uptake and L-malic acid production (Ji et al. 2018; Zhang et al. 2025). These results provide an excellent starting point for further studies on the mechanisms of glycerol transport as well as the interaction of the Glp transporters in *A. niger*.

## Supplementary information

Below is the link to the electronic supplementary material.

ESM 1 (PDF 1.68 MB)

## Data Availability

All data supporting the findings of this study are available within the paper and its Supplementary Information.
