# Supplementary material for: Characterization of three glycerol transporters with broad sugar specificities from Aspergillus niger
Source: Appl Microbiol Biotechnol. 2026 May 8;110(1):199. doi: 10.1007/s00253-026-13854-6 (PMC13323096; doi:10.1007/s00253-026-13854-6)
Supplement: Supplementary file 1 — (PDF 1.68 MB) [file 253_2026_13854_MOESM1_ESM.pdf]

Supplementary Material

Characterization of three glycerol transporters with broad sugar specificities from *Aspergillus niger*

Liinu Nummela[1,2], Christina Lyra[1,2], Leena Pitkänen[1], Henry N. Maina[3], Miia R. Mäkelä[1,2*]

[1]Department of Bioproducts and Biosystems, Aalto University, Espoo, Finland

[2]Department of Microbiology, University of Helsinki, Helsinki, Finland

[3]Department of Food and Nutrition, University of Helsinki, Helsinki, Finland

*Corresponding author: Miia R. Mäkelä, email: miia.makela@aalto.fi

Journal: Applied Microbiology and Biotechnology

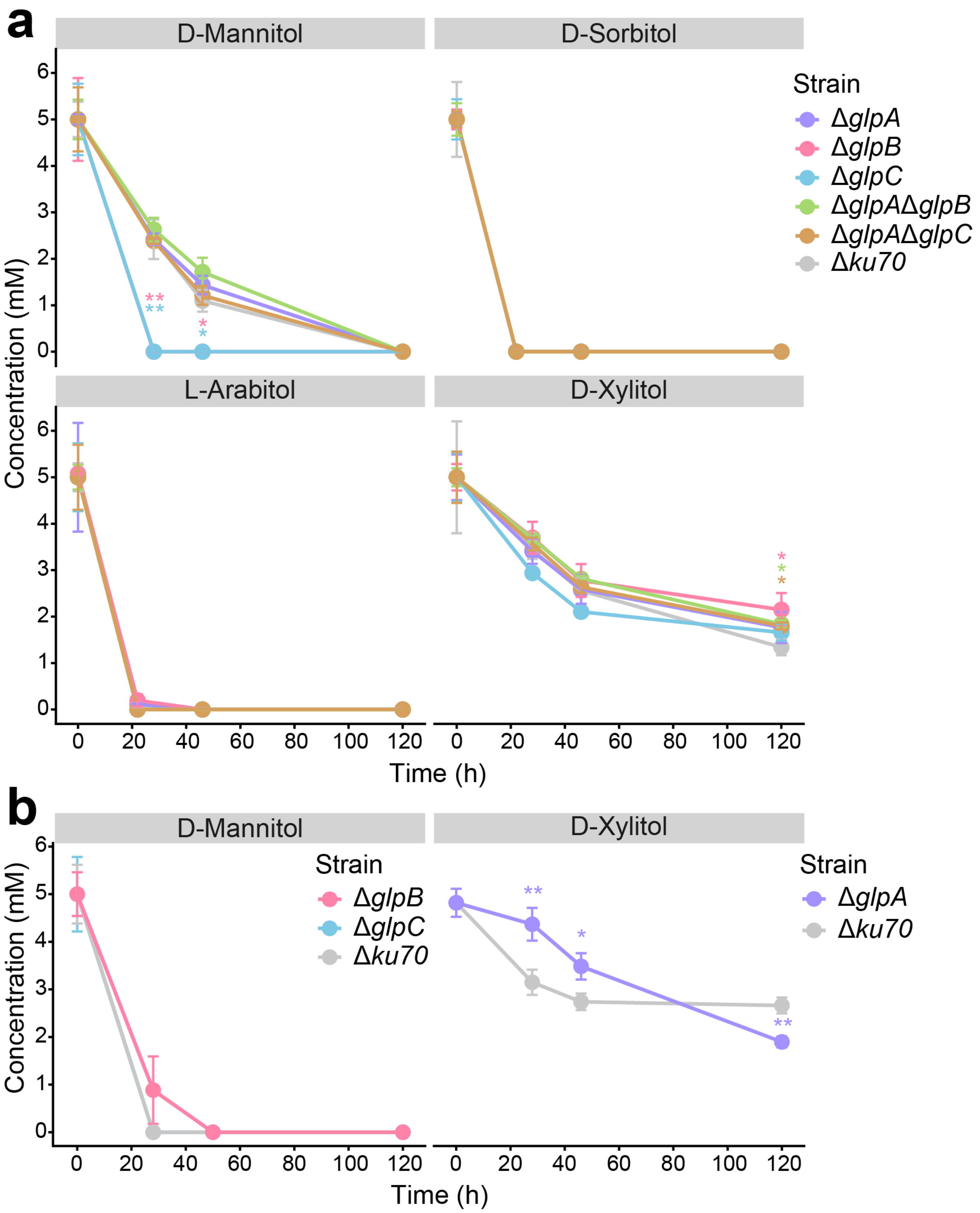

Fig. S1 Polyol consumption of the *Aspergillus niger glp* deletion mutants and the reference strain. a) Time courses for Δ*glpA* (purple), Δ*glpB* (pink), Δ*glpC* (blue), Δ*glpA*Δ*glpB* (green), Δ*glpA*Δ*glpC* (orange) and Δ*ku70* reference strain (grey) in liquid media supplemented with 5 mM D-mannitol, D-sorbitol, L-arabitol or D-xylitol. b) Time courses of an independent polyol

consumption assay for Δ*glpB*, Δ*glpC* and Δ*ku70* in liquid media supplemented with 5 mM D-mannitol, and Δ*glpA* and Δ*ku70* in liquid media supplemented with 5 mM D-xylitol. Liquid cultures were sampled over time and polyol concentrations in the supernatants were analyzed using HPAEC-PAD. Because experiments were conducted in separate runs, all data was normalized using a correction factor determined as the ratio of the starting polyol concentration and the measured polyol concentration at 0 h for each experiment. Error bars represent standard deviations from biological triplicates. Statistical significance was assessed using an unpaired, two-tailed Welch's *t*-test with un-equal variances at each timepoint (* P-value ≤ 0.05, ** P-value ≤ 0.01).

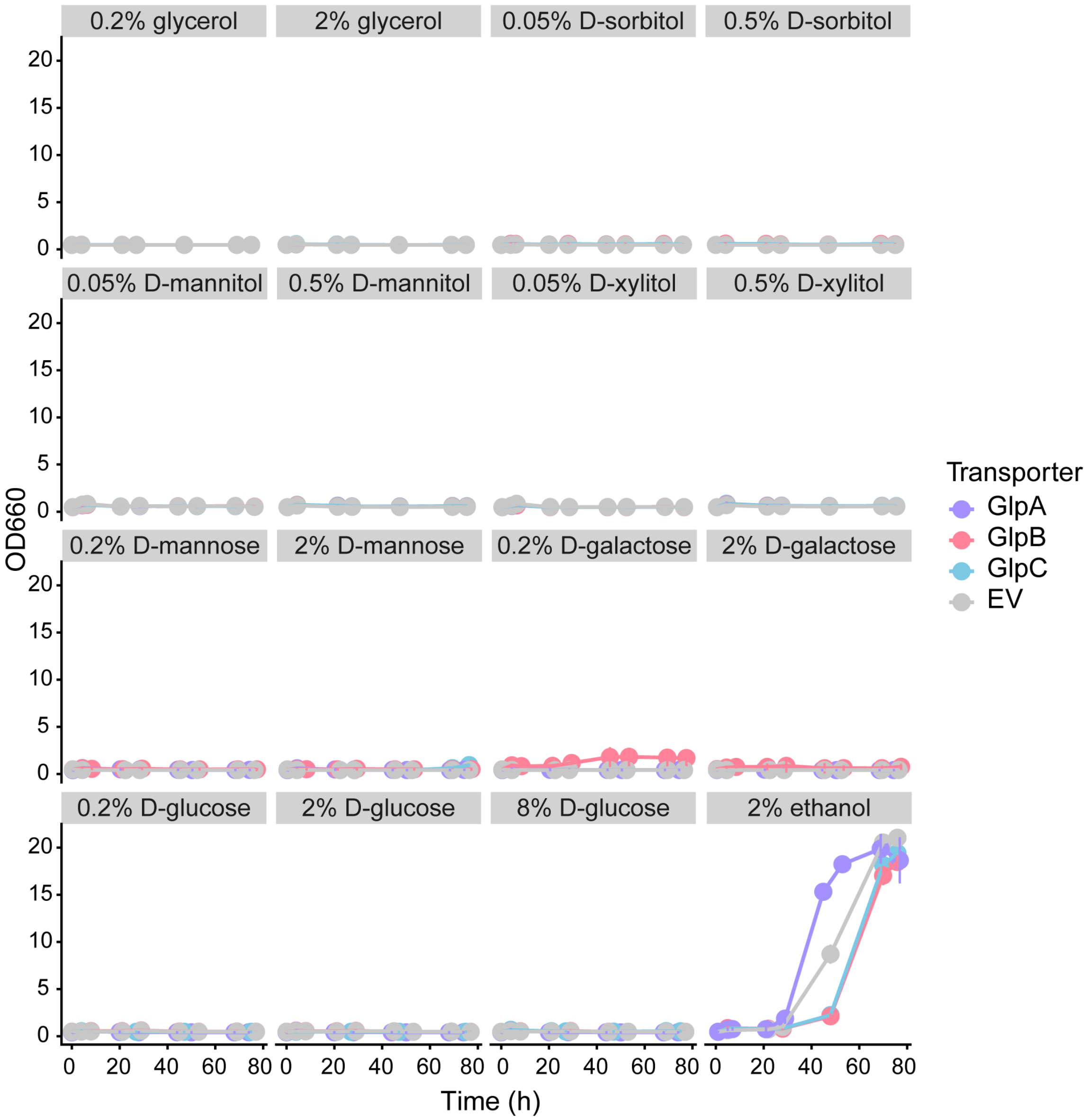

Fig. S2 Liquid growth experiment of Saccharomyces cerevisiae IMK1010 heterologously expressing glpA (purple), glpB (pink) or glpC (blue) from Aspergillus niger. The strains were grown in synthetic medium supplemented with various sugars and polyols: 0.2% or 2% glycerol; 0.05% or 0.5% D-sorbitol, D-mannitol or D-xylitol; 0.2% or 2% D-mannose or D-galactose; 0.2%, 2% or 8% D-glucose. A strain expressing empty pUDE453 vector (EV; grey) was used as a negative control for sugar uptake. Ethanol served as a control carbon source for growth. Error bars represent standard deviations from biological triplicates.

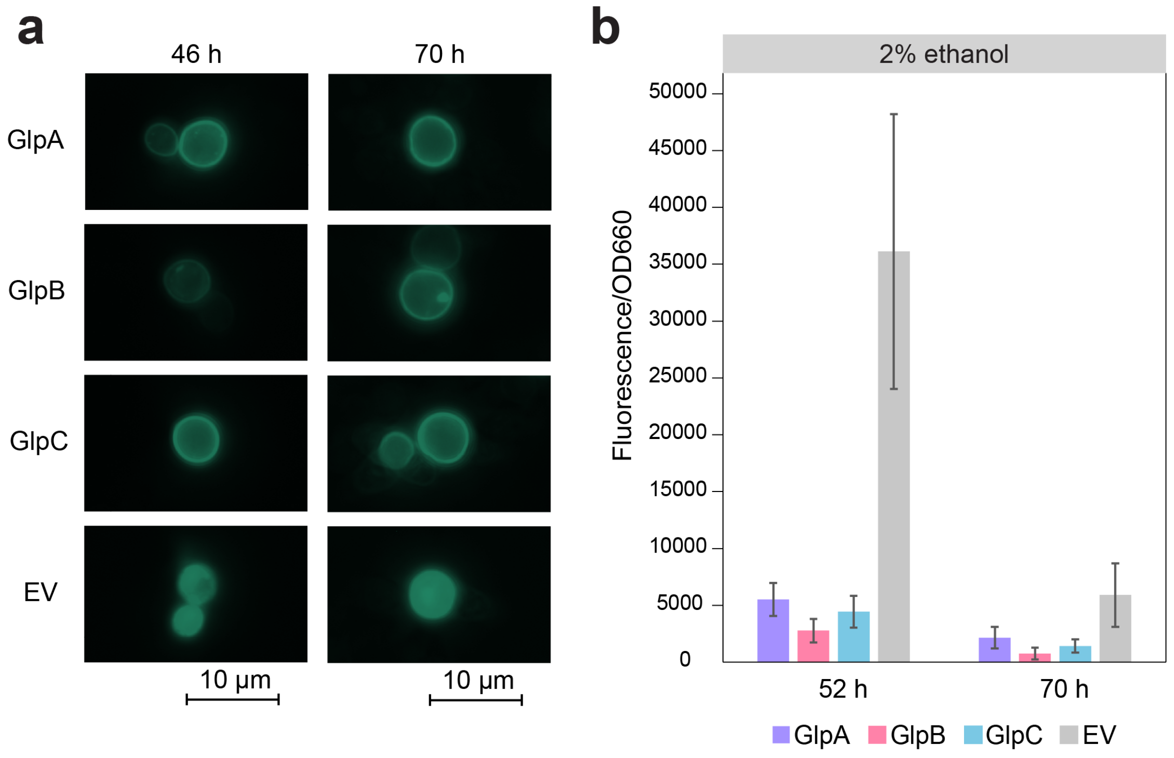

Fig. S3 GlpA, GlpB and GlpC production levels of different *Aspergillus niger glp* expressing *Saccharomyces cerevisiae* IMK1010 strains**.** a) Fluorescence images showing the signal ranges measured with consistent exposure of heterologously produced GlpA, GlpB and GlpC on 2% ethanol after 46 h and 70 h. A strain expressing an empty pUDE453 vector (EV) was used as a negative control. Scale bar = 10 µm. b) OD660 normalized GFP fluorescence (exc/em 510/540 nm) of the 2% ethanol liquid cultures grown 52 h and 70 h. Error bars represent standard deviations from biological triplicates. No statistical significance between the Glp-producing *S. cerevisiae* strains was observed when using an unpaired, two-tailed Welch's *t*-test with un-equal variances at each timepoint. EV = empty vector.

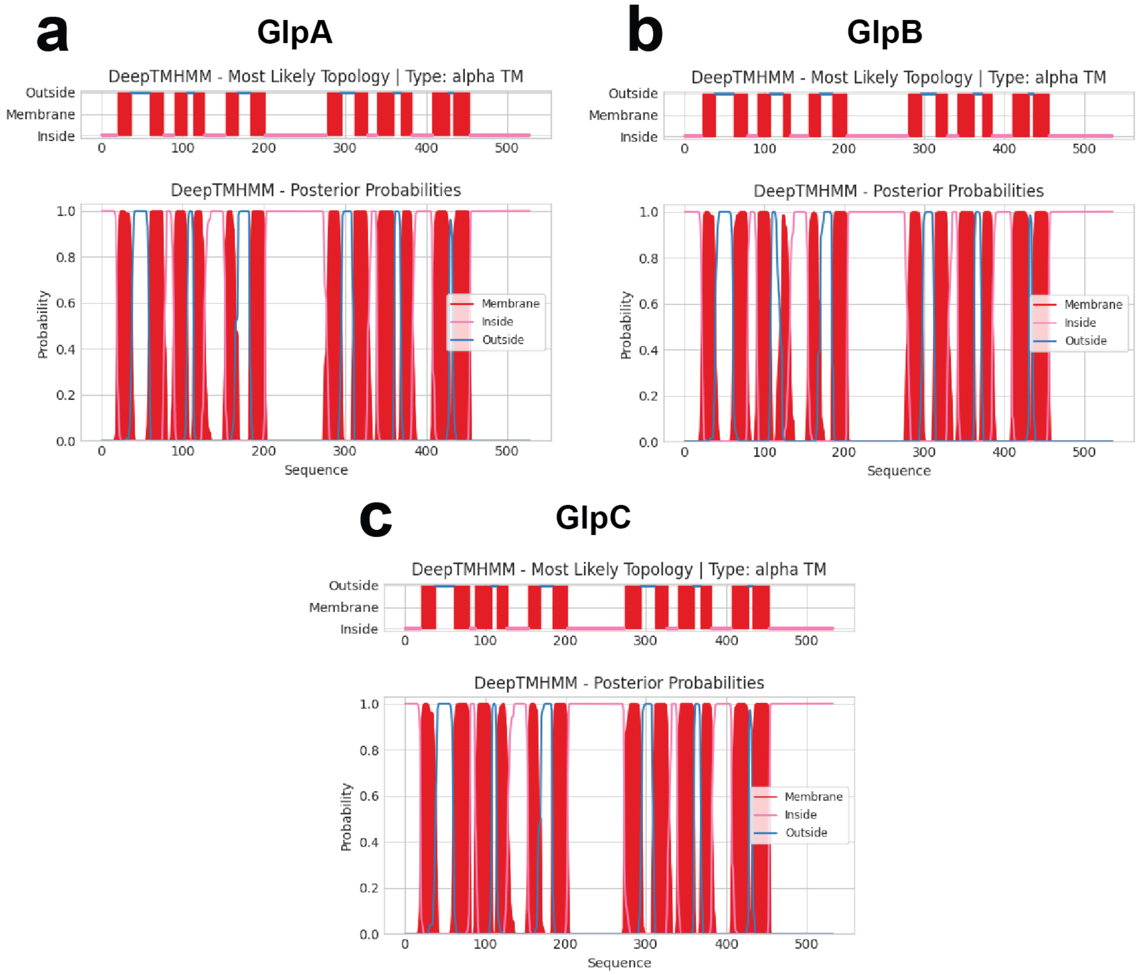

Fig. S4 Topology analysis of *Aspergillus niger* a) GlpA, b) GlpB and c) GlpC using DeepTMHMM v1 (Hallgren et al. 2022). In the subfigures, the upper graph illustrates the predicted transmembrane regions, including transmembrane helices, and intracellular and extracellular domains, while the lower graph visualizes the probability of each region along the sequence.

Table S1. Plasmids used in this study.

Plasmid	Relevant genotype	Source
pTwist_*glpA*	*Aspergillus niger 235*[a]	This study
pTwist_*glpB*	*A. niger 817*[a]	This study
pTwist_*glpC*	*A. niger 935*[a]	This study
pTwist_*hxt5*	*Saccharomyces cerevisiae hxt5*	This study
pUDE453	2 µm ampR *URA3 pTEF1-mal11-GFP-tCYC1*	Marques et al. 2018
pUDE453 empty	2 µm ampR *URA3 pTEF1-GFP-tCYC1*	This study
pUDE453 *hxt5*	3 µm ampR *URA3 pTEF1-hxt5-GFP-tCYC1*	This study
pUDE453 *glpA*	2 µm ampR *URA3 pTEF1-235-GFP-tCYC1*	This study
pUDE453 *glpB*	2 µm ampR *URA3 pTEF1-817-GFP-tCYC1*	This study
pUDE453 *glpC*	2 µm ampR *URA3 pTEF1-935-GFP-tCYC1*	This study
ANEp8 *cas9* LIC	AMA1 ampR *pyrG cas9 ptRNAPro1 ttRNA*	Song et al. 2018
gRNA *glpA*	AMA1 ampR *pyrG cas9 ptRNAPro1 235 ttRNA*	This study
gRNA *glpB*	AMA1 ampR *pyrG cas9 ptRNAPro1 817 ttRNA*	This study
gRNA *glpC*	AMA1 ampR *pyrG cas9 ptRNAPro1 935 ttRNA*	This study

[a]Gene numbers were retrieved from the JGI MycoCosm portal (https://mycocosm.jgi.doe.gov/Aspni_NRRL3_1.home.html).

Table S2. Primers and guide RNA sequences used in this study.

Primer ID	Direction[a]	Sequence (5' to 3')[b]	Purpose[c]
p453_bb_fw	F	GGAGGGGAAAATTTATATT	sGA
p432_453_bb_rv	R	GGATCCACTAGTTCTAGAAA	sGA
p432_453_flank_fw	F	GCAATCTAATCTAAGTTTTC	sGA
p453_flank_rv	R	TTCACCTTTAGAACCTTGAA	sGA
TEF1_prom_fw	F	AGGGTGTCGTTAATTACCCG	cse, csc
CYC1_termin_rv	R	CCTTCCTTTTCGGTTAGAGCG	cse, csc
235_An_1_5F	F	TTCTTTTCTTTTTCCGCTGGTT	RT, csa
235_An_2550_3F	F	**actgctaggattcgctatcg**ACGCTTTTTGATACCTGCTACG	RT
235_An_n_447_5F	F	GCGTTGGCTCCAGTCTTGT	RT
235_An_700_5R	R	**cgatagcgaatcctagcagt**TGTGAAAAGATGCTATACCGAGA	RT
235_An_3050_3R	R	GTTCCCACTTGGCCTATCGG	RT
235_An_n_2728_3R	R	AGTGTTTGAATTCTGTCACGCA	RT, csa
817_An_175_5F	F	ACCGAGCATAAGTGGGTGG	RT, csa
817_An_2476_3F	F	**actgctaggattcgctatcg**TGGTTAGATTAGATGGTGTGTCC	RT
817_An_n_450_5F	F	TTCATTTCTCCCCGTTCATTC	RT
817_An_675_5R	R	**cgatagcgaatcctagcagt**TCAGGACTGATGCAGGTCG	RT
817_An_2976_3R	R	GGGGTGTCGTTTACTGGAC	RT
817_An_n_2756_3R	R	TTGCGTCTAGTATGAGGGTG	RT, csa
935_An_190_5F	F	GGATCTAGTGAACCCTGTGTG	RT, csa
935_An_2485_3F	F	**actgctaggattcgctatcg**TATCACACTGCAACCTTCCA	RT
935_An_n_440_5F	F	GGCTAAGACGCTTCCAATCT	RT
935_An_690_5R	R	**cgatagcgaatcctagcagt**GATCAAACCAAACAGCGTTGA	RT
935_An_2985_3R	R	TTCCGTTAGTCTTCTAATAGCCT	RT
935_An_n_2736_3R	R	TCGAGGCTGCTCAACAAC	RT, csa
235_An_1690_P3	R	CCGTCATGGTCTCCCACATCGACGAGCTTACTCGTTTCG	gRNA
235_An_1690_P4	F	GATGTGGGAGACCATGACGGGTTTTAGAGCTAGAAATAGCAAG	gRNA
817_An_883_P3	R	TGATCATGATGGTGGCACCGACGAGCTTACTCGTTTCG	gRNA
817_An_883_P4	F	GGTGCCACCATCATGATCATGTTTTAGAGCTAGAAATAGCAAG	gRNA
935_An_964_P3	R	CGGTACTTGATCGCCCACGGGACGAGCTTACTCGTTTCG	gRNA
935_An_964_P4	F	CCGTGGGCGATCAAGTACCGGTTTTAGAGCTAGAAATAGCAAG	gRNA

[a]F: forward, R: reverse.

[b]The 20 bp guide RNA sequences used for targeted gene deletions are shown in red font. The linker is shown in bold lowercase font.

[c]sGA: Synthetic gene amplification; cse: colony screening *Escherichia coli*; csc: colony screening *Saccharomyces cerevisiae*; RT: Rescue Template; csa: colony screening *Aspergillus niger;* gRNA: Single guide RNA

References

Hallgren J, Tsirigos KD, Pedersen MD, Almagro Armenteros JJ, Marcatili P, Nielsen H, Krogh A, Winther O (2022) DeepTMHMM predicts alpha and beta transmembrane proteins using deep neural networks. bioRxiv https://doi.org/10.1101/2022.04.08.487609

Marques WL, Mans R, Henderson RK, Marella ER, ter Horst J, de Hulster E, Poolman B, Daran JM, Pronk JT, Gombert AK, van Maris AJA (2018) Combined engineering of disaccharide transport and phosphorolysis for enhanced ATP yield from sucrose fermentation in *Saccharomyces cerevisiae*. Metab Eng 45:121–133. https://doi.org/10.1016/j.ymben.2017.11.012

Song L, Ouedraogo JP, Kolbusz M, Nguyen TTM, Tsang A (2018) Efficient genome editing using tRNA promoter-driven CRISPR/Cas9 gRNA in *Aspergillus niger*. PLoS ONE 13(8):e0202868. https://doi.org/10.1371/journal.pone.0202868
